# Evaluation of imaging performance of major image guidance systems

**DOI:** 10.2349/biij.7.2.e11

**Published:** 2011-04-01

**Authors:** MF Chan, J Yang, Y Song, C Burman, P Chan, S Li

**Affiliations:** 1 Memorial Sloan-Kettering Cancer Center, New York, NY 10065, USA; 2 Monmouth Medical Center, Long Branch, NJ, 07740, USA; 3 Temple University Hospital, Philadelphia, PA, 19140, USA

**Keywords:** Cone beam CT, MVCT, tomotherapy, image quality, IGRT

## Abstract

**Purpose:**

The imaging characteristics of two popular kV cone-beam CT (CBCT) and two MVCT systems utilised in image-guided radiation therapy (IGRT) were evaluated.

**Materials and methods:**

The study was performed on Varian Clinac iX, Elekta Synergy S, Siemens Oncor, and Tomotherapy. A CT phantom (Catphan-504, Phantom Laboratory, Salem, NY) was scanned for measurements of image quality including image noise, uniformity, density accuracy, spatial resolution, contrast linearity, and contrast resolution. The measurement results were analysed using in-house image analysis software. Reproducibility, position correction, and geometric accuracy were also evaluated with markers in a smaller alignment phantom. The performance evaluation compared volumetric image properties from these four systems with those from a conventional diagnostic CT (CCT).

**Results:**

It was shown that the linearity of the two kV CBCT was fairly consistent with CCT. The Elekta CBCT with half-circle 27-cm FOV had higher CT numbers than the other three systems. The image noises of the Elekta kV CBCT, Siemens MV CBCT, and Tomotherapy fan-beam CT (FBCT) are about 2–4 times higher than that of the Varian CBCT. The spatial resolutions of two kV CBCTs and two MV CBCTs were 8-11 lp/cm and 3-5 lp/cm, respectively.

**Conclusion:**

Elekta CBCT provided a faster image reconstruction and low dose per scan for half-circle scanning. Varian CBCT had relatively lower image noise. Tomotherapy FBCT had the best uniformity.

## INTRODUCTION

Image-guided radiation therapy (IGRT) is an emerging radiation treatment modality. A key advantage of IGRT, as compared to conventional radiation treatment techniques, is its ability to maximally spare critical organs and localise the target through correlation of the pre-treatment images with planning images. Conceptually, IGRT consists of two distinct processes: image-guided target delineation and image-guided dose delivery. Several techniques have been developed for IGRT in recent years, with cone-beam CT (CBCT) being one of the most popular and accurate methodologies. The prominent features in CBCT are the acquisition of volumetric images at radiotherapy machines with patients in their treatment positions and the ability to adjust the patient treatment location by correlating the target, critical organs and/or fiducial markers between daily setup CBCT and conventional planning CT (CCT). Thus, intensity-modulated radiation therapy (IMRT) can be delivered with better accuracy of target localisation.

Several major radiation oncology vendors have released their state-of-the-art CBCTs. In the past few years, there have been some studies [1–3] on the comparisons of flat-panel imager-based MV versus kV CBCT, or Tomotherapy MVCT versus helical kV CT. However, since there has been no systematic study to compare their performance, it is not surprising that radiation oncology centres often have difficulties in selecting a suitable CBCT technique for their IGRT treatment delivery [4]. This study attempted to systematically evaluate the imaging performance of two popular kV CBCT and two MVCT systems for IGRT implementation. The goal was to provide an objective assessment and comparison of these widely used CBCT systems.

## MATERIALS AND METHODS

**Figure 1:**
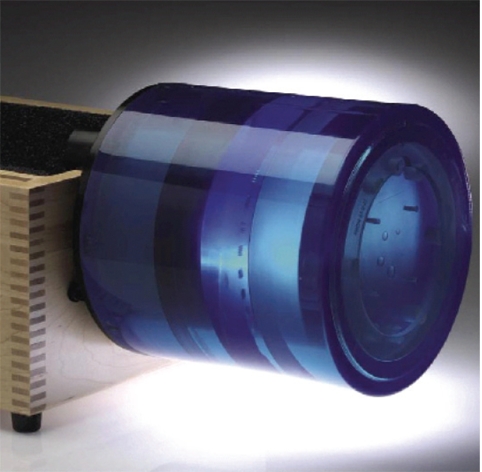
Catphan-504 is designed to evaluate the performance of axial and spiral CT scanners with enhanced sensitometry samples for radiation therapy planning. Its modular design includes test modules CTP404 (slice geometry & contrast linearity), CTP528 (high resolution), CTP515 (low contrast), and CTP486 (uniformity) modules.

The study was performed on Varian Cl-iX CBCT, Elekta XVI kV CBCT, Siemens Oncor MV CBCT, and Tomotherapy Hi-Art Helical MV fan-beam CT (FBCT) systems. The performance evaluation involved comparison of volumetric image data acquired from these four systems with a conventional CT scanner (Philips Brilliance Big Bore). A phantom (Catphan-504, Phantom Laboratory, Salem, NY), shown in Figure 1, was used to characterise image quality in terms of image noise, uniformity, density accuracy, spatial resolution, contrast linearity and contrast resolution. The phantom was scanned using pre-selected parameters and cross-referenced for constancy checks. A 27-cm field of view (FOV) was used for all CBCT scans except for the Tomotherapy FBCT system, which uses a fixed 40-cm FOV. In-house image analysis software developed by Memorial Sloan-Kettering Cancer Center (MSKCC) was used for the image analysis. All imaging systems were accepted and commissioned prior to their use in clinics and quarterly preventive maintenance was done by the manufacturer’s engineers thereafter. Physicists also carried out quality assurance (QA) routinely according to the established protocols [5,6] to ensure that the imaging systems were at their best performance.

### Operational descriptions of CBCT systems

#### Varian kV CBCT

The On-Board Imager (OBI) system consists of a kV X-ray source (KVS) and a kV amorphous-silicon digital imaging detector (KVD) mounted on the linear accelerator using robotic arms (ExactTM), which are orthogonal to the electronic portal imaging device (aSi-1000, PortalVisionTM, Varian Medical Systems). The raw images can be acquired by rotating the linac gantry over 360o for 660 projections (or frames) with a typical setting of 125 kV, 80 mA, and 25 ms. There are two modes of image acquisition. In the “full-fan” mode used for small anatomic sites such as the head and neck, the centre of the KVD is aligned with the isocentre in the longitudinal-lateral plane and 50 cm away from the isocentre in the vertical direction, while the reconstructed volumetric FOV is a cylinder of 25.6 cm in diameter and 16 cm in length. If the required FOV in the transverse plane is larger than 25.6 cm, the “half-fan” acquisition mode is used with the image panel offset laterally by 14.6 cm. The clinical protocols of CBCT call for the use of a “bowtie” filter for better image quality and lower skin dose. The “full bowtie” and “half bowtie” filters are used for full and half-fan scans, respectively. Both filters are made of aluminum with 1.5-mm thickness in the centre. The user has the option to create new imaging protocols or modify the existing ones by changing imaging geometry, the X-ray techniques, and the reconstruction parameters. In this study, the parameters were 2.5-mm slice thickness, with the full-fan and half-fan modes of 24-cm and 27-cm FOV, respectively. Both scanning modes were used in this study.

#### Elekta kV CBCT

The X-ray Volume Imaging (XVI) system consists of a kV X-ray source and a detector panel mounted orthogonally to the MV portal imager. There are three sizes of FOV: small, medium, and large. Medium and large FOVs are selected by offsetting the centre of the 40 × 40 cm^2^ detector panel to 11.5 cm and 19 cm, respectively, from the central axis of the kV X-ray beam, while the small FOV is obtained by centering the panel. The panel is located 53.6 cm beyond the machine isocentre with FOV ranging from approximately 26 to 52 cm. Collimators are inserted to restrict the kV beam to the FOV and to the longitudinal length being imaged, usually 12.8 or 26.4 cm. Preset parameters are configured per anatomical site for imaging geometry, beam characteristics, and reconstruction method. The parameters include, but are not limited to, tube potential, number of frames, mA and ms per frame, start and stop gantry angles, and reconstruction resolution (1-mm pixel size for medium resolution and 0.5-mm pixel size for high resolution). Commonly used presets were used in this study. Tube potential of 100 kV is used for head and neck, and 120 kV for pelvis and chest. A 200-degree gantry rotation (namely half-circle) with small FOV is used for the head and neck while full rotation with medium FOV is used for the pelvis and chest. In this study, both full-circle and half-circle were used for 27-cm FOV.

#### Siemens MV CBCT

The beam conditions in CBCT mode are fixed by the manufacturer using the low (i.e., 6 MV) X-ray beam of the machine and the amorphous-silicon electronic portal imager (a-Si EPID). The gantry makes a 200° arc rotation around the isocentre from 270° to 110° (International Electrotechnical Commission Convention). Two hundred projection images are acquired on a 40 × 40 cm^2^ a-Si EPID at a source-to-image distance of 145 cm. The field width and length are fixed with a square 27.4 × 27.4 cm^2^ field. Delivery protocols are identified by the nominal number of monitor units (MU) selected by the user (from 2 to 60). In Monmouth Medical Center, the 3, 5, 8, 10, 12, and 15 MU protocols have been used clinically, and the 60 MU protocol is for calibration purposes. The protocol of 8 MU was used in this phantom measurement.

#### Tomotherapy MV FBCT

The imaging process consists of a MV fan-beam CT acquisition. A conventional 6 MV linear accelerator and a detector array system are mounted opposite each other on a ring gantry that continuously rotates during the imaging acquisition while the treatment couch continuously translates through the gantry, thus scanning in a helical pattern. For MVCT imaging, the operation of the linear accelerator is adjusted such that the nominal energy of the incident photon beam is reduced to 3.5 MV. The fan beam is collimated to a length of 0.5 cm and a width of 40 cm at isocentre. Three clinical MVCT acquisition modes (fine, normal, and coarse) are available for (2, 4, and 6 mm) slice thickness, respectively. The slice thickness is determined by the pitch value (longitudinal distance that the couch moves during one gantry rotation). The phantom measurement in this study was imaged in the normal mode. The image reconstruction pixel matrix is defaulted at 512 × 512, and the FOV has a diameter of 40 cm.

#### Image noise and uniformity

Image noise and uniformity were measured by scanning the Catphan-504 phantom in air. On the central slice of the CTP486 module, five square (3.5 × 3.5 cm^2^) regions of interest (ROI) were cropped in the centre and in the peripheries at top, bottom, left, and right of the image. Noise in the image was calculated using the standard deviation of the pixel values divided by the mean values in the ROIs. Uniformity was measured using the spread of the mean values over the five ROIs. Varian half-fan mode and Elekta half-circle scans were used in this comparison.

#### Spatial resolution and contrast resolution

Spatial resolution study was performed by scanning CTP528 module in the same phantom. The resolution insert has a radial design pattern. The 2-mm thick aluminum contrast figures are cast into position on the radial gauge, which has resolution sections ranging from 1 to 21 line pairs (lp) per cm. Streaking artifacts in kV CBCTs are commonly enhanced due to missing projections, beam hardening, and scatter radiation. However, the radial design of the module eliminates the possibility of streaking artifacts from other test inserts. The highest number of visible lp was considered the spatial resolution in lp/cm. The CTP515 module consists of a series of cylindrical rods of various diameters and three contrast levels to measure low contrast performance. Varian full-fan mode and Elekta full-circle scans were used in this comparison.

#### Contrast linearity

Contrast linearity was performed by scanning CTP404 module for all CBCT/FBCT systems. The measurements were done by cropping ROI (5 × 5 mm^2^) from 7 different density-inserts and calculating their average pixel values. The values for the different materials of the CBCT/FBCT systems were compared with those of standard CT numbers from CCT data sets. Varian half-fan mode and Elekta half-circle scans were used in this comparison.

## RESULTS AND DISCUSSION

### Image noise and uniformity

The mean pixel values of five ROIs in water-equivalent medium and their standard deviations were 991 and 7.2, 936 and 13.3, 1061 and 28.2, 1080 and 29.8 for Varian, Elekta, Siemens, and Tomotherapy, respectively. The calculated noise levels were 0.7%, 1.4%, 2.7% and 2.8% for Varian, Elekta, Siemens, and Tomotherapy, respectively. The uniformities for the above four CBCT/FBCT systems, in order, were 0.27%, 0.44%, 3.6%, and 0.26%. For a fair comparison, the calculated values from ROIs on the transverse images should be normalised to the volume of interest (VOI) of 10 mm^3^ since the noises do increase with the mean scan volume of ROIs. In the current study, we did not perform the normalisation because this study attempts to conduct evaluations based on the clinical settings, not a mathematical formalisation. Image reconstruction quality of these four IGRT modalities varies from disease site, reconstruction algorithm, reconstructed slice thickness, and image matrix resolution, etc. Therefore, the results were clinical measures for the introductory comparison, not a full scale detailed imaging analysis.

### Spatial resolution and contrast resolution

**Figure 2:**
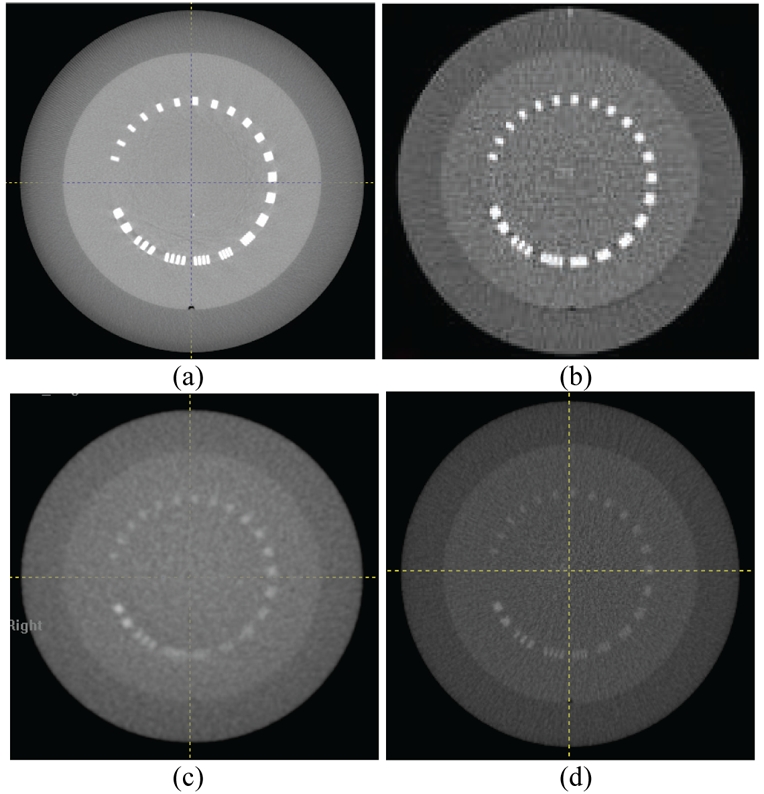
Images of CTP528 module of the Catphan acquired to determine spatial resolution with the four CBCT/FBCT systems; a) Varian kV CBCT; b) Elekta kV CBCT; c) Siemens MV CBCT; d) Tomo MV FBCT.

Figure 2 shows slices of CTP528 module of the Catphan acquired with the four systems. Bars in group 11 were visible, corresponding to 11 lp/cm for Varian CBCT. MV CBCT/FBCT systems have relatively poor spatial resolution (3-5 lp/cm) [7]. However, the difference in spatial resolution between the MV CBCT/FBCT and kV CBCT data sets was small compared to the difference in noise and contrast. This allows the use of fixed small objects in the image registration. These small objects, such as surgical clips, can be helpful in the determination of target location if they are located around a tumour [8]. The use of higher matrix size during reconstruction decreases the pixel size accordingly, thus increasing the spatial resolution; however, it is not used for localisation purposes because of the additional time required to reconstruct the CBCT image. A previous study has shown that the image quality of Siemens CBCT from the 3 MU protocol, 2.5 cGy at isocentre, was sufficient for bony registration, but that a higher dose of 6–10 cGy, typically corresponding to 8 to 16 MU protocols, was necessary to distinguish soft tissue contrast [6]. Another study has shown that 1% contrast resolution (soft tissue contrast) can be resolved by using 3–16 cGy for Siemens MV CBCT images [9]. Good low contrast resolution can be found in both Varian and Elekta CBCT systems: it is evident that circles 8, 5, and 1 for contrast of 1% (smallest diameter 3-mm), 0.5% (smallest diameter 6-mm), and 0.3% (smallest diameter 15-mm) were visible in the images of CTP515 module.

### Contrast linearity/Sensitometry

**Figure 3:**
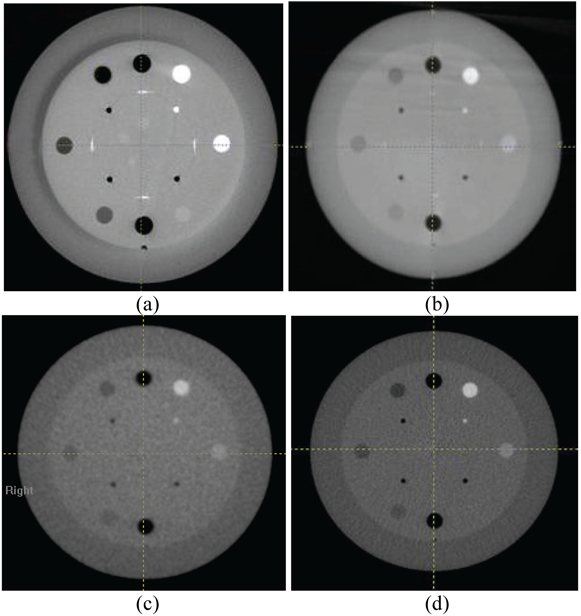
Images of CTP404 module of the Catphan acquired to determine contrast linearity with the four CBCT/FBCT systems; a) Varian kV CBCT; b) Elekta kV CBCT; c) Siemens MV CBCT; d) Tomo MV FBCT.

**Figure 4:**
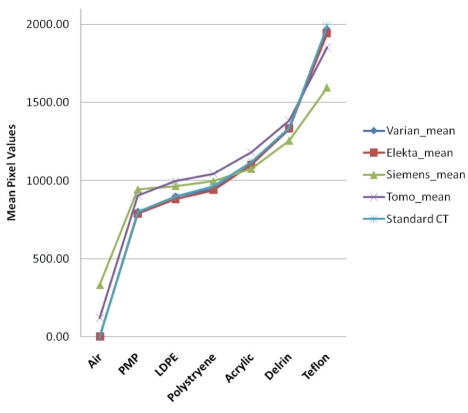
Average pixel values measured with CTP404 module of the Catphan phantom on Varian, Elekta, Siemens, and Tomotherapy versus standard CT for 7 different density materials.

Figure 3 shows slices of CTP404 module of the Catphan acquired with the four systems. There were seven different density-inserts in the module (Air, PMP, LDPE, Polystryene, Acrylic, Delrin, and Teflon). The linearity for the two kV CBCT was fairly consistent with CCT. The Elekta CBCT with half-circle 27-cm FOV had higher CT number variations than the other three modes because Elekta half-circle scan was preset with very low exposure to low scanning doses important for the head and neck scanning. For low-density materials, the CT numbers with Varian CBCT were 300–600 units lower than those of the Elekta CBCT. Those differences would be diminished when full-circle scan was used by Elekta CBCT. Figure 4 shows the mean pixel values of different density-inserts for the CBCT/FBCT systems versus those of CCT. As shown, CT numbers for both kV CBCT systems were very close to those of CCT - within 2% and 3% for Varian and Elekta systems, respectively.

### Discussion

The image quality measurements of the current study included image noise, uniformity, image resolution, and density linearity. The Elekta CBCT image noise of half circle 27-cm FOV scans were higher than that of the Varian CBCT, while the image noise levels in Elekta’s other operational modes, similar to those of Siemens and Tomotherapy CBCT/FBCT systems, were 3–4 times higher than the Varian CBCT. The uniformity of all CBCT/FBCT systems, except Siemens, was better than 1%. The linearity of the two kV CBCT systems was fairly consistent with CCT. The accuracy of all CBCT/FBCT systems was comparable and has no significant difference. Using the same kV X-ray beams, there are differences between the scanning techniques between Varian’s half-fan for large FOV with bowtie filter and full-fan for small FOV versus the Elekta half-circle for small FOV and whole circle for large FOV. Since Siemens and Tomotherapy CT are MV CT images, their spatial resolution and contrast resolution were poor relative to those of the kV CBCT. This is due to inherent blurring for the relatively large size of high energy X-ray detectors in the image panel, as well as more Compton scattering and pair production in the patient with MV beams. Considering the effects of broad-beam scattering on CBCT, one can shorten the scanning distance or narrow cone beam along the longitudinal direction to improve the image quality. Both Varian and Elekta CBCT have the visible “ring” artifacts due to unbalanced projection row data for a large body using a large FOV setting. Bowtie filters can improve the image quality in some cases but not always, depending on the scanning parameters. Based on this investigation with Elekta XVI on phantom, the use of Bowtie filter (F1) can increase the contrast-to-noise ratio (CNR) for the typical scans with 100 kV cone beams, while decreasing the CNR for the typical scans with high energy (138 kV) cone beams. Bad image cells in the flat image panel may cause the ring artifacts if cell response has not been corrected or recalibrated by interpolating neighbouring cells’ data. Dose from kV CBCT and MV CT is in the range of few cGy per scan but could be much lower for the Elekta system because of possible settings of lower mAs and less scan angles. Varian CBCT dose could be lowered with the lower mAs settings. The differences in imaging geometry of these systems may also contribute to the differences in image quality and to the scope of application as well. Clinically, Elekta CBCT can provide larger FOV and longer length along the longitudinal direction than Varian due to its larger flat-panel imager design. The spatial resolution of both Varian and Elekta systems’ acceptance tests are 7 lp/cm (10 lp/cm for updated version XVI Release 4.2 b11 of Elekta CBCT acceptance test protocol), and we can usually observe 11-12 lp/cm at high resolution mode with a full-circle scan. Table 1 compared the clinical properties among the four CBCT systems being studied.

**Table 1:** Comparisons among two kV CBCT and two MV CBCT/FBCT systems.

	**Varian**	**Elekta**	**Siemens**	**Tomotherapy**	**CCT**
**Mechanical Adjustment**					
kV Source Position	< 1 mm	< 1 mm	NA	NA	< 1 mm
kV Detector Position	< 1 mm	< 1 mm	NA	NA	NA
MV Detector Position	< 1 mm	< 1 mm	< 1 mm	< 1 mm	NA
**Image Quality**					
High Resolution	8-11 lp/cm	8-10 lp/cm	4-5 lp/cm	3-5 lp/cm	8-12 lp/cm
Low Contrast	0.3%	0.9%	1%	3%	0.1%
Spatial Linearity	< 1 mm	< 1 mm	< 1 mm	< 1 mm	< 1 mm
**Resolution & Geometry**					
Sensitometry	< 2%	< 3%	< 20%	< 13%	< 0.5%
Average Pixel Values	991+7.2	936+13.3	1061+28.2	1080+29.8	999.6+4.0
Image Noise	0.7%	1.4%	2.7%	2.8%	0.4%
Uniformity	0.27%	0.44%	3.6%	0.26%	0.12%
**Imaging Time**	2-min	2-min	3-min	3-min	< 1-min
**Imaging Dose**	1-4 cGy	0.3-4 cGy	3-16 cGy	1-3 cGy	2-4 cGy

Based on a CBCT QA study conducted by MSKCC [10], variation of OBI isocentre with time was summarised as follows: random isocentre shift was relatively small, but systematic isocentre shifts of 0.4-1.4 mm due to misalignment of OBI were observed. Accurate patient setup using OBI requires monitoring of systematic errors in OBI isocentre, such as misalignment with radiation isocentre, gantry rotation and translational motion of the imager. Isocentre shifts and CBCT numbers for OBI were stable over time, as observed by the same group as well as in a previous study [11]. Both kV CBCT uniformity and linearity were comparable to CCT within 2-3%. In general, CCT using more accurate filtered-backprojection reconstruction (FBPR) and more rigid configuration of the X-ray tube, fan-beam collimation, and detectors has superior contrast with less noise, less streaking and ring artifacts as compared to CBCT.

From our annual CBCT dose measurements, the CBCT dose for half-fan beam is about 1 cGy at isocentre and the peripheral dose is about 3 cGy, whereas CT simulator dose at isocentre is about 1 cGy for 35-cm diameter body phantom measurement. As for low contrast resolution, CCT contrast is superior to kV CBCT, and kV CBCT is superior to MV CBCT/FBCT. kV CBCT has pronounced ring artifacts and more noise than CCT due to a less accurate imaging hardware alignment and reconstruction algorithm. Clinically, at a dose of 2–3 cGy, the Tomotherapy MV FBCT images are of sufficient quality for verification of treatment setup, but low contrast object may not be perceptible with MV energies due to the relatively poor signal-to-noise ratio (SNR) performance [9,12,13]. Siemens MV CBCT has poor soft tissue contrast and higher dose to patients. In order to maintain comparable doses for MVCT and kVCT, the number of MV photons incident on the patient must be considerably reduced and this reduction decreases the SNR ratio [9]. Unlike kV CBCT systems, the presence of high atomic number (Z) materials such as tooth fillings or implanted markers did not result in visible metal artifacts for MV CBCT systems [14].

Although contrast linearity of MV CBCT/FBCT imaging is not as close to CCT as the kV CBCT is, MV CBCT/FBCT is superior in its linear relationship between relative electron density and CT number for dose calculation [15]. Because artifacts due to metal objects and beam hardening are less critical for MV sources, MV CBCT/FBCT scans have been used to complement CCT scans when these artifacts are severe [15]. The two MVCT systems utilise the same X-ray sources for both imaging and treatment and hence provide more accurate geometrical information than the kV CBCT systems although the kV CBCT systems provide better quality images [4]. The isocentre accuracies of Tomotherapy and Siemens were reported to be approximately 0.2 mm and 0.5 mm, respectively, in all directions, which were confirmed by their isocentre accuracy testing.

## CONCLUSION

This study confirmed the comparability of imaging performance of four popular clinically available CBCT/FBCT systems. Our objective evaluation of imaging performance revealed that the volumetric data rendered by the four CBCT/FBCT systems are accurate, as compared with conventional CT. In a summary, Elekta CBCT provided faster image reconstruction, Varian CBCT had relatively lower image noise, and Tomotherapy had the best uniformity.
